# Proposal of Allobaileyella gen. nov., Allobaileyella intestinalis comb. nov., Allolucifera gen. nov., Allolucifera butyrica comb. nov., Allomicrovenator gen. nov., Allomicrovenator marinus comb. nov. and Allomicrovenatoraceae fam. nov. as replacement names for Baileyella, Baileyella intestinalis, Lucifera, Lucifera butyrica, Microvenator, Microvenator marinus and Microvenatoraceae, respectively

**DOI:** 10.1099/ijsem.0.007061

**Published:** 2026-02-03

**Authors:** Umakant Bhoopati Deshmukh, Marko Kostovski, Aharon Oren

**Affiliations:** 1 Department of Botany,, Institution of Higher Learning, Research and Specialized Studies Centre (affiliated to Gondwana University, Gadchiroli), Janata Mahavidyalaya, Chandrapur 442 401, India; 2 University Ss Cyril and Methodius, Faculty of Medicine, Institute of Microbiology & Parasitology, Ul. 50-ta Divizija br. 6, 1000 Skopje, Republic of North Macedonia; 3 Department of Plant and Environmental Sciences, The Institute of Life Sciences, The Hebrew University of Jerusalem, Edmond J. Safra Campus, Jerusalem 9190401, Israel

**Keywords:** *Baileyella*, illegitimate names, *Lucifera*, *Microvenator*

## Abstract

The prokaryotic genus names *Baileyella* Wylensek *et al*. 2021, *Lucifera* Sánchez-Andrea *et al*. 2019, and *Microvenator* Wang *et al*. 2022 are illegitimate because they are later homonyms of *Baileyella* Özdikmen 2009 (a fossil member of the Gonyaulacaceae, Gonyaulacales, Dinophyceae), *Lucifera* Baker 2010 (Keroplaridae, Diptera) and *Microvenator* Ostrom 1970 (a fossil member of the Caetagnatidae, Saurichia, Reptilia), respectively [Principle 2 and Rule 51b(5) of the International Code of Nomenclature of Prokaryotes (ICNP)]. We therefore propose the replacement names *Allobaileyella* gen. nov., *Allobaileyella intestinalis* comb. nov., *Allolucifera* gen. nov., *Allolucifera butyrica* comb. nov., *Allomicrovenator* gen. nov., *Allomicrovenator marinus* comb. nov. and *Allomicrovenatoraceae* fam. nov.

The prokaryotic genus *Baileyella* (*Anaerovoracaceae*, *Peptostreptococcales, Clostridia*, *Bacillota*) was described by Wylensek *et al*. in 2020 [1]. The genus currently contains a single species with a validly published name: *Baileyella intestinalis* Wylensek *et al*. 2021 [1,2]. The generic name *Baileyella* is a homonym of *Baileyella* Özdikmen 2009, a fossil member of the Gonyaulacaceae*,* Gonyaulacales*,* Dinophyceae [3]. Based on Principle 2 and Rule 51b(5) of the International Code of Nomenclature of Prokaryotes (ICNP) [4] and the version in effect at the time of publication [5], the name *Baileyella* proposed for a prokaryotic genus is illegitimate.

The ICNP offers two possibilities to resolve the illegitimacy of a name: by replacing an illegitimate name with a new legitimate one or by addressing a request for an opinion to the Judicial Commission to obtain an exception allowing the illegitimate name to be conserved (see [6,7] for recent examples). As the replacements proposed here are not expected to cause errors or confusion, we prefer the first option. In accordance with Rule 54, we here propose replacement names, conserving the meaning of the illegitimate names as originally published.

## Description of *Allobaileyella* Deshmukh, Kostovski and Oren gen. nov.

*Allobaileyella* (Al.lo.bai.ley.el’la. Gr. masc. adj. *allos*, other; N.L. fem. n. *Baileyella*, a bacterial genus; N.L. fem. n. *Allobaileyella*, another *Baileyella*).

Earlier illegitimate homotypic synonym: *Baileyella* Wylensek *et al*. 2021 [1,2].

The description of the genus is as given for *Baileyella* [1].

The type species is *Allobaileyella intestinalis* (Wylensek *et al*. 2021) Deshmukh *et al*.

## Description of *Allobaileyella intestinalis* (Wylensek *et al.* 2021) Deshmukh, Kostovski and Oren comb. nov.

*Allobaileyella intestinalis* (in.tes.ti.na’lis. N.L. fem. adj. *intestinalis*, pertaining to the gut).

Illegitimate basonym: *Baileyella intestinalis* Wylensek *et al*. 2021 [2].

The description of the species is as given for *Baileyella intestinalis* [1].

The type strain is RF-744-FAT-WT-3^T^ (=DSM 106896^T^=JCM 34418^T^), isolated from the faeces of a 4-month-old pig in Kranzberg, Bavaria, Germany.

The 16S rRNA gene sequence accession number of the type strain is MN537520.

The genome sequence of the type strain can be accessed from GenBank as GCF_048191505.1.

The prokaryotic genus *Lucifera* (*Sporomusaceae*, *Selenomonadales, Negativicutes*, *Bacillota*) was described by Sánchez-Andrea *et al*. in 2018 [8]. The genus currently contains a single species with a validly published name: *Lucifera butyrica* Sánchez-Andrea *et al*. 2019 [8,9]. The generic name *Lucifera* is a homonym of *Lucifera* Baker 2010 (Keroplaridae, Diptera) [10]. Based on Principle 2 and Rule 51b(5) of the ICNP [4], which is retroactive, the name *Lucifera* proposed for a prokaryotic genus is illegitimate. In accordance with Rule 54, we here propose replacement names, conserving the meaning of the illegitimate names as originally published.

## Description of *Allolucifera* Deshmukh, Kostovski and Oren gen. nov.

*Allolucifera* (Al.lo.lu.ci’fe.ra. Gr. masc. adj. *allos*, other; N.L. fem. n. *Lucifera*, a bacterial genus; N.L. fem. n. *Allolucifera*, another *Lucifera*).

Earlier illegitimate homotypic synonym: *Lucifera* Sánchez-Andrea *et al*. 2019 [8,9].

The description of the genus is as given for *Lucifera* [8].

The type species is *Allolucifera butyrica* (Sánchez-Andrea *et al*. 2019) Deshmukh *et al*.

## Description of *Allolucifera butyrica* (Sánchez-Andrea *et al.* 2019) Deshmukh, Kostovski and Oren comb. nov.

*Allolucifera butyrica* (bu.ty’ri.ca. N.L. neut. n. *acidum butyricum*, butyric acid; N.L. fem. adj. *butyrica*, pertaining to butyric acid).

Illegitimate basonym: *Lucifera butyrica* Sánchez-Andrea *et al*. 2019 [8,9].

The description of the species is as given for *Lucifera butyrica* [8].

The type strain is ALE^T^ (=DSM 27520^T^=JCM 19373^T^), isolated from sediments of an acid rock drainage environment, Tinto River, Spain.

The 16S rRNA gene sequence accession number of the type strain is HG317002.

The genome sequence of the type strain can be accessed from GenBank as GCF_900476375.1.

The prokaryotic genus *Microvenator* (*Microvenatoraceae*, *Bradymonadales*, *Deltaproteobacteria*, *Pseudomonadota*) was described by Wang *et al*. in 2022 [11]. The genus currently contains a single species with a validly published name: *Microvenator marinus* Wang *et al*. 2022 [11]. The generic name *Microvenator* is a homonym of *Microvenator* Ostrom 1970, a fossil member of the Caetagnatidae, Saurichia, Reptilia [12]. Based on Principle 2 and Rule 51b(5) of the ICNP [4], which is retroactive, the name *Microvenator* proposed for a prokaryotic genus is illegitimate. In accordance with Rule 54, we here propose replacement names, conserving the meaning of the illegitimate names as originally published.

## Description of *Allomicrovenator* Deshmukh, Kostovski and Oren gen. nov.

*Allomicrovenator* (Al.lo.mi.cro.ve.na’tor. Gr. masc. adj. *allos*, other; N.L. masc. n. *Microvenator*, a bacterial genus; N.L. masc. n. *Allomicrovenator*, another *Microvenator*).

Earlier illegitimate homotypic synonym: *Microvenator* Wang *et al*. 2022 [11].

The description of the genus is as given for *Microvenator* [11].

The type species is *Allomicrovenator marinus* (Wang *et al.* 2022) Deshmukh *et al*.

## Description of *Allomicrovenator marinus* (Wang *et al.* 2022) Deshmukh, Kostovski and Oren comb. nov.

*Allomicrovenator marinus* (ma.ri’nus. L. masc. adj. *marinus*, of the sea, marine).

Illegitimate basonym: *Microvenator marinus* Wang *et al*. 2022 [11].

The description of the species is as given for *Microvenator marinus* [11].

The type strain is V1718^T^ (=KCTC 72082^T^=MCCC 1H00380^T^), isolated from coastal sediment, Xiaoshi Island, PR China.

The 16S rRNA gene sequence accession number of the type strain is MZ234079.

The genome sequence of the type strain can be accessed from GenBank as GCA_007993755.1.

## Description of *Allomicrovenatoraceae* Deshmukh, Kostovski and Oren fam. nov.

*Allomicrovenatoraceae* (Al.lo.mi.cro.ve.na.to.ra.ce’ae. N.L. masc. n. *Allomicrovenator*, type genus of the family; -*aceae*, ending to denote a family; N.L. fem. pl. n. *Allomicrovenatoraceae*, the *Allomicrovenatoraceae* family).

Earlier illegitimate homotypic synonym: *Microvenatoraceae* Wang *et al*. 2022 [11].

The description of the family is as given for the family *Microvenatoraceae* [11].

The type genus is *Allomicrovenator* Deshmukh *et al*.

## Abbreviations

ICNP: International Code of Nomenclature of Prokaryotes
